# Uncommon clinical presentation of cystic fibrosis in a patient homozygous for a rare CFTR mutation: a case report

**DOI:** 10.1186/s12887-020-1980-y

**Published:** 2020-02-27

**Authors:** Joanna Jaworska, Aleksandra Marach-Mocarska, Dorota Sands

**Affiliations:** 1 Institute of Mother and Child, Cystic Fibrosis Department, ul. Kasprzaka 17A, 01-211 Warsaw, Poland; 2 Children’s Memorial Health Institute, Department of Gastroenterology, Hepatology, Feeding Disorders and Pediatrics, Al. Dzieci Polskich 20, 04-730 Warsaw, Poland

**Keywords:** Cystic fibrosis, CF, Metabolic acidosis, Case report, Genetic testing

## Abstract

**Background:**

Cystic fibrosis (CF) is the most common, life-threatening, autosomal-recessive disorder among Caucasians. To date, approximately 2000 mutations in the CFTR gene have been reported. Some of these mutations are very rare, and some represent individual sequence changes in the gene. The introduction of newborn screening (NBS) in high prevalence countries for CF has considerably changed the diagnosing of this metabolic disease. Currently, in most cases, a diagnosis is made based on NBS, including or expanded with DNA analysis and confirmed with sweat chloride tests, rather than waiting until the child has already developed signs and symptoms. However, in rare cases, NBS does not provide enough information to confirm or reject a CF diagnosis. Not only are there small groups of patients who have false-negative or false-positive NBS results, but there is also a growing number of patients with positive NBS results in whom results of sweat tests and genetic examinations do not provide definite conclusions. Despite all knowledge and modern diagnostic tools at our disposal, sometimes the clinical presentation is so inconclusive, that making a final diagnosis remains a challenge.

**Case presentation:**

In this case report, we present a male infant of Polish origin, whose symptoms and laboratory findings (including metabolic acidosis) were strongly suggestive of metabolic disease other than cystic fibrosis. Newborn screening for CF was positive, but the first sweat test results were equivocal, and initial and extended molecular tests were negative. Finally, after considering broad differential diagnosis, introducing treatment specific for CF and excluding other metabolic diseases, a third expanded genetic test revealed the presence of a rare pathogenic mutation in both alleles of the CFTR gene: c.4035_4038dupCCTA (p.Ser1347ProfsX13).

**Conclusion:**

Although CF is considered a monogenic disorder, the relationship between genotype and phenotype is very complex. The reported case shows the unusual presentation of the disease. The patient’s clinical symptoms and laboratory findings, in combination with molecular test results, provide useful information for further observing the genotype-phenotype correlations in cystic fibrosis.

## Background

Cystic fibrosis is the most common life-limiting, autosomal-recessive disorder among Caucasians [1]. To date, approximately 2000 mutations in the CFTR gene on chromosome 7 have been reported. Some of them are very rare, and some represent individual sequence changes in the gene. A wide range of mutation testing methods are available: from specific mutation detection based on the well-known spectrum in a defined population (these tests are being widely used in newborn screening programs) to mutation screening methods, like the sequencing of the entire coding region of the CFTR gene (which are being used to identify rare and novel mutations).

The introduction of newborn screening in high prevalence countries for CF has considerably changed the diagnostic process of this metabolic disease. Screening protocols vary among different countries, but the measurement of immunoreactive trypsinogen (IRT) in the first week of life remains the initial test of all NBS programs across the world [2]. The second tier of testing is required because only a minority of children with a raised ITR will have CF. This can be either to repeat the IRT measurement or to perform DNA analysis. The scope of molecular tests used among countries also varies – from checking for a single pathologic mutation (F508del as the most common) to DNA panels of an increasing number of CFTR mutations, to the complete gene sequencing.

Currently, in most cases, CF diagnosis is made based on NBS, including or expanded with DNA analysis and confirmed with sweat chloride tests, rather than on already developed signs and symptoms (Table 1). Thus, diagnosis is easy and relatively quick, but pediatricians are obliged to inform parents of outwardly healthy newborns or young infants about a life-threatening disease. In rare cases, NBS does not provide enough information to confirm or reject a CF diagnosis. There is a group of patients, who have false-negative NBS results (3–5% of tested newborns) – some who exhibit meconium ileus within the first days of their lives (in these cases the diagnosis is often made when the NBS results are still pending), some who present later with other suggestive symptoms, some who are diagnosed through family screening, and some who have atypical (nonclassic) CF. In most children with false-positive NBS results, the diagnosis is relatively quickly refuted – they are either healthy or healthy carriers of a CFTR mutation. However, in rare cases, particularly in unwell preterm infants, excluding CF remains challenging. It should also be noted that advanced molecular techniques are not always followed by clinical knowledge (i.e., there are identified CF mutations, for which phenotypic consequences remain unclear). Therefore, recognition of children with an equivocal diagnosis is an implication of all screening protocols, but especially those, which are followed by extended gene analysis. There is a growing group of patients “labeled” as CF SPID (CF Screen-Positive Inconclusive Diagnosis), in whom, in the absence of clinical symptoms, results of sweat tests and genetic examinations do not provide definite conclusions [3]. This group of patients consists of two subgroups: children with a normal sweat chloride level and with two recognized CFTR mutations, one of which has unclear phenotypic consequences, and children with repeatedly intermediate sweat chloride levels with one or no mutations. Despite all knowledge and all modern diagnostic tools at our disposal, sometimes the clinical presentation is so inconclusive, that making a final diagnosis remains a challenge.

**Table 1:** Signs and symptoms of CF (bolded – present in the reported patient)

	**Common respiratory**	**Common non-respiratory**	**Less common**
age-independent	▪ productive cough ▪ **respiratory infection** with typical CF pathogen	▪ salty-tasting skin	
neonatal		▪ meconium ileus ▪ abdominal cramps ▪ fatty stools	▪ **protracted jaundice** ▪ intestinal atresia ▪ **fat-soluble vitamin deficiency**
infancy	▪ **chronic cough** ▪ recurrent wheeze ▪ **recurrent lower respiratory tract infections**	▪ **failure to thrive** due to exocrine pancreatic insufficiency with steatorrhoea, diarrhea, and abdominal distension	▪ rectal prolapse ▪ **anaemia, oedema and hypoproteinemia** ▪ pseudo-Bartter’s syndrome ▪ hypochloremic metabolic alkalosis ▪ **cholestasis**

## Case presentation

**Fig. 1:**
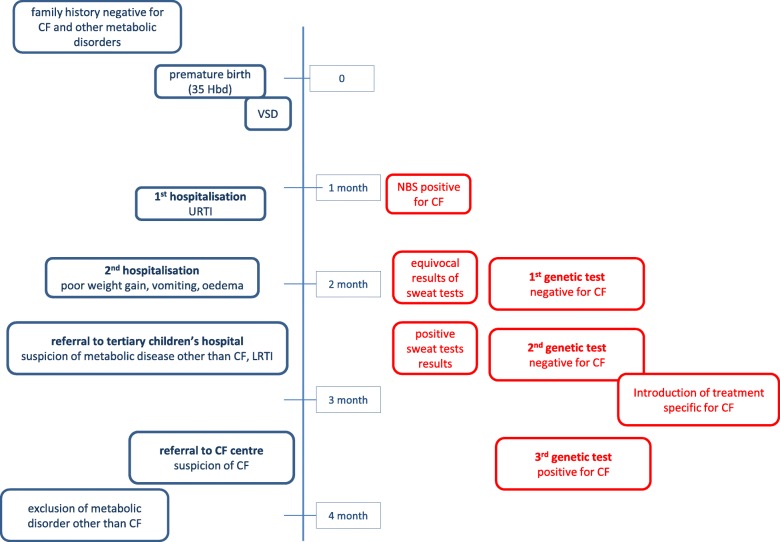
Timeline (legend: CF - cystic fibrosis, VSD – ventricular septal defect, URTI – upper respiratory tract infection, LRTI – lower respiratory tract infection)

We present the case of a one-year-old patient of Polish origin, who was referred to our Clinic at the age of 4 months (timeline – Fig. 1).

The boy was born prematurely (35 weeks of gestation) from a pregnancy complicated by maternal pneumonia, hypothyroidism and cervical insufficiency. In the neonatal period, the child was treated with phototherapy due to jaundice and diagnosed with ventricular septal defect. Family history was positive for allergy (older brother) and celiac disease (uncle). He was hospitalised for the first time due to an upper respiratory tract infection in the second month of life. Further hospitalisation was necessary 2 weeks later due to poor weight gain, intense regurgitation and vomiting. The physical examination revealed: a poor general condition and general nutrition status, pale skin, peripheral oedema, signs of mild dehydration, a systolic murmur and an umbilical hernia. Abnormal laboratory findings included: anaemia (red cell concentrate transfusion was needed), hypoproteinemia, elevated conjugated bilirubin, GGTP, lactate and ammonia, and decreased alpha-1-antitrypsin level (Table 2). Newborn screening was positive for cystic fibrosis (IRT over 99.4th percentile). However, sweat test results (conductivity) were equivocal – of the 4 outcomes, one was positive and three proved negative (Table 2), and the first genetic examination excluded 700 mutations. The abdomen ultrasound examination did not reveal any abnormalities, while the radiographic barium studies showed gastroesophageal reflux and excluded congenital anomalies of the digestive tract. Cranial sonography was negative. Due to insufficient oral food administration, the boy was fed through a nasogastric tube. Because of the symptoms of bronchitis, antibiotic and aerosol therapy were introduced. The child was then referred to a tertiary children’s hospital with the suspicion of metabolic disease.

**Table 2:** Crucial laboratory findings – second hospitalisation (abnormalities - bolded)

Test name	Result	Normal range
**haemoglobin**	**7.4 g/dl**	8.7–16.9 g/dl
**red blood cells**	**2.62 M/μl**	3.8–5.8 M/μl
**total protein**	**3.4 g/dl**	5.7–8.9 g/dl
**total bilirubin**	**2.48 mg/dl**	0.1–1.3 mg/dl
**conjugated bilirubin**	**0.68 mg/dl**	0–0.20 mg/dl
**GGTP**	**469 U/l**	12–122 U/l
**lactate**	**52.5 mg/dl**	4.5–19.8 mg/dl
**ammonia**	**90.2 μg/dl**	17–79.9 μg/dl
**alpha-1-antitrypsin**	**58 mg/dl**	90–200 mg/dl
sweat conductivity	▪ **105 mmol/l** ▪ 33 mmol/l ▪ 51 mmol/l ▪ 16 mmol/l	negative: < 60 mmol/l equivocal: 60–80 mmol/l positive: > 80 mmol/l

### Pulmonological problems

On admission, the child presented with cough, rales and rhonchi. The chest X-ray revealed bilateral perihilar opacities. The aspirate of the respiratory tract discharge culture was positive for *Staphylococcus aureus*, *Stenotrophomonas maltophilia* and *Candida lusitaniae*. The boy was treated with levofloxacin and micafungin according to the sensitivity test results. Although control cultures proved negative and auscultatory findings subsided, the control chest X-ray did not show significant improvement.

### Gastrointestinal and nutritional problems

The bile acids and GGTP concentrations were elevated. The alpha-1-antitrypsin level was not decreased this time (Table 3). There were vitamin A and E deficiencies, while vitamin D concentration was average. The labial frenulum was undercut, but on discharge, there was still a need for a nasogastric tube, due to ineffective sucking.

**Table 3:** Crucial laboratory findings – tertiary children’s hospital **(abnormalities - bolded)**

Test name	Result	Normal range
**bile acids**	**32 μmol/l**	0–10 μmol/l
**GGTP**	**654 U/l**	0–203 U/l
alpha-1-antitrypsin	1.09 g/l	0.9–2.0 g/l
**vitamin A**	**156.9 ng/ml**	200.0–800.0 ng/ml
**vitamin E**	**1.7** μg**/ml**	3.8–16.0 μg/ml
vitamin D	60.2 pg/ml	25.1–154.0 pg/ml
**total protein**	**36.4 g/l**	51.0–73.0 g/l
**albumin**	**24.3 g/l**	38.0–54.0 g/l
**haemoglobin**	**9.3** → **8**.**0 g/dl**	9.5–13.0 g/dl
**red blood cells**	**3.48** → **3.19 M/μl**	3.8–5.0 M/μl
**total bilirubin**	**2.48 mg/dl**	0.1–1.3 mg/dl
**conjugated bilirubin**	**0.54 mg/dl**	0–0.20 mg/dl
blood gas test		
**pH** **HCO3** pCO2	**7.29** **19.6 mmol/l** 45.3 mmHg	7.35–7.43 22.0–26.0 mmol/l 45.0–50.0 mmHg
**lactate**	**28.4 mg/dl**	4.5–19.8 mg/dl
**ammonia**	**131 μg/dl**	20–80 μg/dl
TSH	3.6 μIU/ml	0.4–7.0 μIU/ml
fT4	1.1 ng/dl	0.6–1.4 ng/dl
**sweat test - pilocarpine iontophoresis**	▪ **101.6 mmol/l**	positive > 50 mmol/l
	▪ not enough sweat	

### Metabolic, hematological and neurological problems

The boy presented with recurrent oedema (hypoproteinemia, hypoalbuminemia) and needed repeated intravenous albumin supplementation. Due to progressing anaemia, a second red cell concentrate transfusion was necessary. The blood gas test showed metabolic acidosis. The lactate and ammonia levels were elevated (Table 3). Due to the generalised diminished muscle tone and tendon reflexes, and the suspicion of metabolic disease, the following examinations were performed: ophthalmoscopy (negative), head MRI (dilation of left Sylvian fissure), and EMG (negative). Although the serum amino acids and gas chromatography/mass spectrometry of urine metabolites (GC/MS) revealed several small irregularities, it did not create a pattern of a specific metabolic disease. Hypothyroidism and galactosemia were excluded. Control ammonia and lactate levels remained within normal ranges, while the GC/MS was pending. The sweat chloride test (pilocarpine iontophoresis) was performed on both upper limbs. There was not enough sweat collected on one arm, but the other proved positive (Table 3). Expanded genetic testing for CF did not show any pathological mutation. Due to a strong suspicion of CF, a hyperenergetic, high-protein diet and pancreatic enzyme replacement therapy were introduced and the patient was referred to our Centre.

At the time of admission, the boy presented in good general condition, although his nutritional status remained unsatisfactory. The physical examination revealed: generalised diminished muscle tone, an umbilical hernia, a nasogastric tube, a systolic murmur and isolated bilateral rhonchi and rales. Sweat tests were repeated using two methods – the classic one (pilocarpine iontophoresis) and conductometric – both results were confirmatory (116 mmol/l, positive results ≥60 mmol/l and 76 mEq/l, positive results ≥60 mEq/l). The faecal elastase test was positive (< 15 μg/g, normal range > 200 μg/g), confirming an exocrine pancreatic insufficiency. Following repeated negative GC/MS results, a final metabolic consultation excluded metabolic defects other than CF. The third expansion of molecular testing finally revealed identical pathogenic mutations in two alleles of the CFTR gene – c.4035_4038dupCCTA (p.Ser1347ProfsX13). Both of the patient’s parents were diagnosed to be asymptomatic carriers of the mutation. They denied consanguinity. Molecular examinations in the two older siblings of the patient were negative.

After several months of holistic treatment, the child achieved 25 percentile both for body mass (started deep below 3rd pc) and for body length (started from 10th pc) and attained consecutive developmental milestones.

## Discussion and conclusion

### Why was the road to diagnosis so winding?

#### Atypical clinical presentation

Failure to thrive and respiratory infections are typical for CF (Table 1) [4]. Whereas, it should be highlighted that even these typical symptoms are not pathognomonic. Peripheral oedema and anaemia demanding several red cell concentrate transfusions are less common signs (Table 1), but intense regurgitation and vomiting, postural asymmetry, diminished muscle tone and tendon reflexes are not recognized as a part of clinical presentation of the cystic fibrosis disease (Table 4).

**Table 4:** Symptoms and laboratory findings suggestive of metabolic disease other than CF

Symptoms	Laboratory findings
intense regurgitation and vomiting	↑ ammonia
umbilical hernia	↑ lactate
postural asymmetry	↓alpha-1-antitrypsine
generalized diminished muscle tone	metabolic acidosis
diminished tendon reflexes	GC/MS irregularities
	serum amino acids irregularities

#### Laboratory findings suggestive of a different metabolic disease

Elevated lactate and ammonia levels, irregularities in GC/MS and in serum amino acids, and metabolic acidosis, which were observed, are strongly suggestive of a different metabolic disease (Table 4). Acidosis in CF occurs exceptionally rarely, usually as a result of bicarbonate stool loss. It can also be caused by increased dietary acid load due to a high-protein diet and pancreatic enzyme supplementation, but neither of these concerns this case [5].

#### Equivocal sweat chloride test results

The causes of a false-positive sweat test results were taken into consideration and excluded, i.e.; celiac disease, atopic dermatitis, ectodermal dysplasia, fucosidosis, G6PD deficiency, glycogenosis type 1, mucopolysaccharidosis, adrenal, thyroid and parathyroid insufficiency, pseudohypoaldosteronism, nephrosis, familial cholestasis, diabetes insipidus. Malnutrition can cause both false-positive and false-negative results. False-negative results may also be induced by peripheral oedema, technical issues, glucocorticosteroid therapy and some mutations (p.e. 3849 + 10kbC → T). Sweat test measurements performed in better nutritional status and without peripheral oedema were highly positive in this patient.

#### Negative results of the first two genetic tests

The first two molecular tests excluded 86% of mutated CFTR gene alleles in the Polish population, with 99% sensitivity. The third molecular workup confirmed the correct diagnosis. C.4035_4038dupCCTA is a mutation that leads to a frameshift and is expected to result in complete loss-of-function of the CFTR protein, which is consistent with the child’s high sweat chlorides, pancreatic insufficiency and recurrent respiratory tract infections. This mutation is sporadic, not found in the CFTR2 Database, but registered in the CFTR and HGMD Databases. Hitherto it has only been reported in patients of Polish origin and only in combination with other mutations [6]. The applied method (DNA sequencing) enables the detection of rare mutations and new changes, which are not registered in databases. This is especially important for patients in East-Central Europe, because of the high heterogeneity of the population [7].

## Conclusion

 Although CF is considered a monogenic disorder, the relationship between genotype and phenotype is very complex. The reported case shows the unusual presentation of the disease. The patient’s clinical symptoms and laboratory findings, in combination with molecular test results, provide useful information for further observation of genotype-phenotype correlations in cystic fibrosis.

It should be noted, that among the strengths of this case report, is the fact that the patient was diagnosed and treated in a central tertiary children’s hospital as well as in a tertiary cystic fibrosis centre with personnel specifically trained in metabolic diseases and cystic fibrosis, supported by advanced diagnostic infrastructure. Thus, all steps of the diagnostic process were undertaken according to current medical knowledge and international guidelines. The limitations include the questionable character of observed signs and symptoms (Table 4), which can be associated with both the rare CFTR mutation accompanied by the atypical clinical presentation, and the prematurity accompanied by the poor general condition of the patient. Also, no firm conclusion can be drawn from a single case.

## Data Availability

The datasets used and analyzed during the current report are available from the corresponding author (JJ) on request.

## Abbreviations

CF SPID: Cystic Fibrosis Screen-Positive Inconclusive Diagnosis
CF: Cystic fibrosis
CFTR: Cystic fibrosis transmembrane conductance regulator
DNA: Deoxyribonucleic acid
EMG: Electromyography
fT4: Free T4 – free thyroxine
G6PD: Glucose-6-phosphate dehydrogenase
GC/MS: Gas chromatography/mass spectrometry
GGTP: Gamma-glutamyl transpeptidase
HCO3: Bicarbonate ion
IRT: Immunoreactive trypsinogen
LRTI: Lower respiratory tract infection
MRI: Magnetic resonance imaging
NBS: Newborn screening
pc: Percentile
pCO2: Partial pressure of carbon dioxide
pH: Decimal logarithm of the reciprocal of the hydrogen ion activity
TSH: Thyroid-stimulating hormone
URTI: Upper respiratory tract infection
VSD: Ventricular septal defect
